# Immunotherapy for Prostate Cancer: Lessons from Responses to Tumor-Associated Antigens

**DOI:** 10.3389/fimmu.2014.00191

**Published:** 2014-05-06

**Authors:** Harm Westdorp, Annette E. Sköld, Berit A. Snijer, Sebastian Franik, Sasja F. Mulder, Pierre P. Major, Ronan Foley, Winald R. Gerritsen, I. Jolanda M. de Vries

**Affiliations:** ^1^Department of Tumor Immunology, Radboud Institute for Molecular Life Sciences, Radboud University Medical Center, Nijmegen, Netherlands; ^2^Department of Medical Oncology, Radboud University Medical Center, Nijmegen, Netherlands; ^3^Juravinski Hospital and Cancer Centre, Hamilton, ON, Canada

**Keywords:** immunotherapy of cancer, prostate cancer, tumor-associated antigens, CRPC, immunotherapy

## Abstract

Prostate cancer (PCa) is the most common cancer in men and the second most common cause of cancer-related death in men. In recent years, novel therapeutic options for PCa have been developed and studied extensively in clinical trials. Sipuleucel-T is the first cell-based immunotherapeutic vaccine for treatment of cancer. This vaccine consists of autologous mononuclear cells stimulated and loaded with an immunostimulatory fusion protein containing the prostate tumor antigen prostate acid posphatase. The choice of antigen might be key for the efficiency of cell-based immunotherapy. Depending on the treatment strategy, target antigens should be immunogenic, abundantly expressed by tumor cells, and preferably functionally important for the tumor to prevent loss of antigen expression. Autoimmune responses have been reported against several antigens expressed in the prostate, indicating that PCa is a suitable target for immunotherapy. In this review, we will discuss PCa antigens that exhibit immunogenic features and/or have been targeted in immunotherapeutic settings with promising results, and we highlight the hurdles and opportunities for cancer immunotherapy.

## Introduction

Prostate cancer (PCa) is the most commonly diagnosed non-cutaneous cancer among men in the United States and is the second leading cause of death from cancer in men (1). In Europe, PCa is also the cancer type with the highest incidence in men apart from skin cancer, while it is the third most common type of cancer after lung cancer and colorectal cancer (2). PCa is usually diagnosed in men above 65 years of age. Depending on the severity of the disease, current treatment options for PCa consist of active surveillance, prostatectomy, radiation therapy, hormonal therapy, or chemotherapy. Up to one-third of patients with a localized tumor eventually fails on local therapy and progress to advanced-stage or metastatic PCa within 10 years. For advanced PCa, androgen deprivation therapy is the standard of care. Although the majority of patients initially respond, most tumors become resistant to primary hormonal therapy within 14–30 months (3). For men with metastatic castration-resistant prostate cancer (mCRPC), the median survival in phase III studies range from 15 to 19 months. For several years, the chemotherapeutic drug docetaxel was the only treatment option for mCRPC, resulting in a median overall survival benefit of 2–3 months compared with the previous treatment regimes mitoxantrone and prednisone (4–6). However, new agents targeting the androgen signaling pathway, immunotherapeutic options, radium-223 treatment, and the new chemotherapeutic treatment modality taxane cabazitaxel are emerging therapies with the ability to improve both the survival and the quality of life.

In 2010, the first cellular immunotherapy was approved as a treatment for mCRPC by the US Food and Drug Administration (FDA). More recently, cancer immunotherapy hit a new peak, Science Magazine elected cancer immunotherapy the breakthrough of 2013 (7). Especially, modulation of T-cell checkpoints via immune checkpoint inhibiting [anti-cytotoxic T lymphocyte antigen-4 (CTLA-4) monoclonal antibodies and anti-programed death (ligand) 1 (PD-(L)1] monoclonal antibodies has been successful. Instead of tacking of the brake of the immune system, as is the case with checkpoint inhibitors, another challenge is out there: enhancement of immune responses to tumor-specific antigens. In this review, we discuss tumor antigens expressed by PCa, how they can be used to combat PCa via immunotherapy, and which hurdles need to be addressed and overcome. Other new treatment modalities are beyond the scope of this study.

## Inflammatory Responses in the Prostate

Inflammation is an innate response to harmful stimuli, such as infections, tissue damage, or tissue malfunction (8, 9). The main goal with the inflammatory process is to clear the potential threat and restore tissue homeostasis. This normally occurs in two phases – the recognition and elimination phase and the resolution and repair phase (8, 10). If the acute response fails in eliminating the inflammatory agents, the inflammation shifts toward a chronic state. Instead of initiating the resolution phase, additional macrophages and lymphocytes are recruited and, depending on the inflammatory inducer, act to remodel the local microenvironment to adapt to an altered tissue homeostasis.

Cancer has been described as a wound that refuses to heal (11), and today many cancers have been tightly correlated with preceding inflammatory responses (12, 13). Several lines of evidence support the theory that inflammation also precedes PCa (9). Proliferative inflammatory atrophy lesions are areas in the prostate with an increased infiltration of inflammatory cells. These regions can merge with prostatic intraepithelial neoplasia, which is considered to be a risk factor for the development of PCa (14, 15). Also, a correlation with regular intake of non-steroidal anti-inflammatory drugs and reduced PCa risk has been observed (16–18).

## Autoimmunity and PCa

Inflammatory response inducers in prostate vary from infections to life style factors, such as diet or smoking (19). Symptomatic prostatitis caused by bacterial infection has been correlated with an increased risk of PCa development (20, 21). However, the causing agents of the majority of symptomatic and asymptomatic prostatitis are not well characterized and are probably multifaceted events (22).

Several studies have reported autoimmune responses against both seminal proteins (23, 24) and prostate antigens causing prostatitis to become chronic (25–27). These finding are additionally verified in animal models, where a cytotoxic cellular response seems to be driving the autoimmune reaction (28). Androgen ablation in patients with PCa is shown to induce high levels of T-cell infiltration into both benign and cancerous prostate sites, indicating that autoimmune responses against prostate antigens might be hormonally regulated (29).

## Antigen-Based Cancer Immunotherapy

The increased knowledge of how specific immune responses are evoked and the development of tools to manipulate the immune system have enabled implementation of novel immune-based cancer therapies. The rationale of these immunotherapies is to induce anti-tumor immune responses, decrease tumor-load, and change the course of the disease. Recognition of target antigens by the immune system is crucial. Several types of immunotherapeutics have been developed, such as peptide vaccines, DNA/RNA vaccines, cell-based vaccines, and T-cell modulators. Although improving overall survival is the primary endpoint of most clinical studies, a better understanding of induced T-cell responses, boosting pre-existing immune responses, and the effect of the tumor microenvironment on the T cells is needed to further improve PCa immunotherapy.

Tumor-associated antigens in PCa can be proteins that are present on prostate cells and on their malignant counterparts. Examples are prostate-specific antigen (PSA), prostate-specific membrane antigen (PSMA), and the cancer/testis antigens (CTAs). In a steady state, these antigens are not provoking strong immune responses. Immunosuppressive mechanisms in the prostate microenvironment, such as transforming growth factor (TGF)-β, regulatory T cells (Tregs), or myeloid-derived suppressor cells, will maintain prostate infiltrating lymphocytes in an inactive state (30–32). In addition, PCa cells exploit several mechanisms to enhance immune tolerance (33). Despite the immunosuppressive microenvironment, several immunotherapeutic approaches are able to induce or enhance tumor-specific immune responses. In the following section, potential tumor antigens and their application as immunotherapeutic targets will be discussed. Table 1 provides an overview of the antigens discussed and clinical results of antigen-based immunotherapy trials.

**Table 1:** **Antigens and their immunogenicity in prostate cancer**.

Antigen	Function	Immunogenicity in PCa	Human clinical trials in PCa	Number of patients with PCa
PSA	Serine protease which cleaves high molecular weight proteins into smaller peptides, resulting in the necessary liquification for spermatozoa to swim freely	Stimulates CTLs *in vivo*	Poxviral vaccine PROSTVAC-VF/PSA-TRICOM showed a longer median overall survival when compared to placebo (34)	82 vs. 40 controls
			A phase I trial with a recombinant vaccinia virus expressing PSA (rV-PSA) showed a stable PSA level for at least 6 months in 14 patients (35)	33
		Production of immunosuppressive cytokines	A study with JBT 1001, a recombinant PSA vaccine, showed a T-cell response in eight patients (36)	10
			A study reported a PSA decrease between 6 and 39% compared to baseline in 11 of the treated patients with PSA-loaded DCs (37)	24
PAP	Protein tyrosine phosphatase which enhances the mobility of sperm	Stimulates CTLs *in vivo*	A phase I/II study reported PAP-specific T-cell responses and an increased PSA doubling time for the plasmid DNA accine pTVG-HP PAP when compared to placebo (38)	22
		Elevated in both prostatic hyperplasia and PCa	Three phase III RCTs, of which two showed a significant increase in overall survival (39, 40), and one (41) showed a trend to increase in overall survival for sipuleucel-T compared with placebo	341 vs. 171 placebo (39)
				82 vs. 45 placebo (40)
				65 vs. 33 placebo (41)
PSMA	Folate hydrolase activity	Presented at the cell surface and in the endothelial lumen, the latter promotes integrin signaling	A phase I trial reported a 50% PSA reduction in four patients treated with ^177^lutetium-labeled J591, a radiolabeled monoclonal antibody against PMSA (42)	35
		Highly overexpressed in PCa	A study using an HLA-A2 restricted PMSA peptide (LLHETDSAV) showed neither clinical nor immune responses. The authors concluded that the used PSMA epitope was poorly immunogenic compared with other HLA-A2-presented peptides (43)	12
			A phase II trial with DCs pulsed with PMSA peptides showed a 50% reduction of PSA in nine patients (44)	33
PSCA	Unknown, overexpressed by most PCas	T-cell activation and proliferation	Two vaccination studies in humans with DCs loaded with PSCA alone or in combination with PAP, PSMA, and/or PSA reported that the vaccine was well tolerated and increased both the PSA doubling time and median overall survival of the patients (45, 46)	12 (45) 6 (46)
MUC-1	Limiting the activation of inflammatory responses	T-cell proliferation	A phase I/II trial with DCs loaded with MUC-1 glycopeptide and KLH showed a reduction of PSA rise in six patients. Immune responses to KLH (6/7) and Tn-MUC-1 (5/7) have been detected (47)	7
			Radioimmunotherapy was combined with or without low-dose paclitaxel in patients with mCRPC and breast cancer. In two patients with mCRPC who received m170 (MUC-1 monoclonal antibody) linked to indium-111, a 50% decline in PSA level was shown which lasted 2 months, and two patients described a decrease in bone pain (48)	9
NY-ESO-1	Unknown, expressed in a variety of tumors	CTLs and antibody-mediated responses	In patients with mCRPC, NY-ESO-1 peptides vaccines were tolerable. Among nine patients, vaccinations appeared to slow PSA doubling time, and yielded antigen-specific T-cell responses in six patients (49)	14
			Immunoactivation following an NY-ESO-1 protein-based vaccine combined with CpG showed humoral and cellular immune responses specific for NY-ESO-1 in 12 and 9 of the vaccinated patients, respectively (50)	13
MAGE-A genes	Down-regulates p53 function through histone deacetylase recruitment	Stimulates CTLs *in vivo*	No human clinical trial performed in PCa	
AKAP-4	Binding protein involved in cytoskeletal regulation and organization by affecting cyclic AMP-dependent protein kinase-A	Stimulated CTLs *in vitro*	No human clinical trial performed in PCa	

## Prostate Cancer Antigens

### Prostate-specific antigen

Prostate-specific antigen is a serine protease produced primarily in the epithelial cells lining the acini and ducts of the prostate gland (51–53). Physiologically, PSA is present at high concentrations in the seminal fluid. Its function is to cleave high molecular weight proteins into smaller peptides, which results in liquification of these peptides. This allows the spermatozoa to swim freely (51). Membrane-bound PSA is expressed by most PCa cells. Upon disruption of the prostate gland tissue by cancerous growth, PSA is released into the circulation. There, PSA can interact with several inflammatory cells, including fibroblasts and macrophages, which might cause chronic inflammation (9, 54, 55). PSA serum levels correlate with the extent of disease and are therefore a useful tumor marker, accurately reflecting tumor status and prognostic for clinical outcome. In case of relapse, PSA levels correlate with tumor recurrence (51, 56). Transcription of the PSA gene is positively regulated by the androgen receptor, which can partly explain the decline in PSA levels in response to androgen deprivation therapy (52). However, high PSA levels are also observed in patients with CRPC, due to the acquired ability of the tumor cells to maintain the androgen receptor function even in the androgen-ablated environment (57).

#### PSA as tumor antigen

Cellular autoimmune responses against PSA have been detected in both healthy men and patients suffering from chronic prostatitis (26, 27, 58), suggesting that PSA has immunogenic properties. It has been used as a target antigen in several immunotherapeutic constructs. Hodge et al. used a vector designated TRICOM, containing three co-stimulatory molecules B7-1, ICAM-1 and LFA-3, and a PSA peptide, for T-cell stimulation (59). Using a similar approach, Kantoff et al. studied a combination of PSA-expressing recombinant viral vectors, where treatment with a vaccinia-based priming vector was followed by six booster injections of a fowlpox-based vector (PROSTVAC-VF). In the phase II, randomized controlled trial in patients with mCRPC, no significant difference in progression-free survival was detected between control group and the vaccinated group. However, vaccinated patients had a longer median overall survival, and a better 3-year survival (60). These clinically meaningful results have to be confirmed in an ongoing phase III trial (Table 2).

**Table 2:** **Ongoing trials encompassing antigen-based immunotherapy**.

Antigen	Study design	Trial identifier	Immunologic endpoints
PSA	Phase II trial of PROSTVAC-VF/PSA-TRICOM with docetaxel and prednisone vs. docetaxel and prednisone alone in patients with mCRPC	NCT01145508 (the study is ongoing but not recruiting new patients anymore)	Immune responses before and after docetaxel and PSA-specific immune responses Primary endpoint: overall survival
	Phase II trial with enzalutamide with or without PROSTVAC-VF/PSA-TRICOM in patients with mCRPC	NCT01867333 (ongoing and recruiting trial, estimated completion date June 2016)	Immune response (not further specified) Primary endpoint: to show increase in time to progression
	Phase III study of PROSTVAC-VF/PSA-TRICOM with or without GM-CSF in patients with mCRPC	NCT01322490 (ongoing and recruiting trial, estimated completion date August 2016)	No immunologic endpoints Primary endpoint: overall survival
PAP	Phase II trial of sipuleucel-T with a pTVG-HP DNA vaccine in patients with mCRPC	NCT01706458 (ongoing and recruiting trial, estimated completion date June 2015)	Primary endpoint: immune responses following treatment with sipuleucel-T
	Phase II trial of sipuleucel-T with concurrent or sequential abiraterone acetate plus prednisone in patients with mCRPC	NCT01487863 (active study, not recruiting, estimated completion date June 2015)	Primary endpoint: sipuleucel-T CD54 upregulation
	Phase II trial of sipuleucel-T and ipilimumab given immediately sequential vs. delayed sequential in patients with mCRPC	NCT01804465 (active study, not recruiting, estimated completion date August 2015)	Primary endpoints: safety of both treatment arms and induction of antibody responses by sipuleucel-T, the proportion of patients on each study arm who achieve an immune response to PAP and/or PA2024
	Phase I study of sipuleucel-T and ipilimumab in patients with mCRPC	NCT01832870 (ongoing and recruiting trial, estimated completion date December 2015)	Primary endpoint: antigen-specific memory T-cell response, antigen-specific proliferation and antibody responses against PAP, PA2024 and PHA
	Phase II trial of sipuleucel-T with or without anti-PD-1 monoclonal antibodies and cyclophosphamide	NCT01420965 (ongoing and recruiting trial, estimated completion date December 2017)	Primary endpoints: feasibility and the immune efficacy of sipuleucel-T alone vs. sipuleucel-T plus cyclophosphamide and anti-PD-1 monoclonal antibodies (CT011) on the change in specific immune response
PSMA	Phase I trial of adoptive T-cell transfer targeted to PSMA in patients with mCRPC	NCT01140373 (ongoing and recruiting trial, estimated completion date June 2014)	No immunologic endpoints Primary endpoint: progression-free survival
	Phase II trial of PSMA antibody drug conjugate in patients with mCRPC	NCT01695044 (ongoing and recruiting trial, estimated completion date January 2015)	Primary endpoints: changes in tumor assessments, serum PSA and circulating tumor cells
	Phase II study of prodrug chemotherapy (G-202) which is activated *in situ* by PSMA of PCa cells or within cancer blood vessels of patients with mCRPC	NCT01734681 (study is not yet open for recruitment, estimated completion date January 2015)	Changes in circulating tumor cells and humoral and cell-mediated immunity to PSMA and other known PCa antigens and to track the persistence, accumulation, and migration of genetically retargeted anti-PSMA autologous T cells
			Primary endpoint: safety and tolerability of immunotherapy
	Phase I trial of anti-PSMA designer T cells after non-myeloablative conditioning in patients with mCRPC	NCT00664196 (ongoing and recruiting trial, estimated completion date July 2016)	Pharmacokinetics and pharmacodynamics of the anti-PSMA designer T cells Primary endpoint: the safety of using modified T cells
PSCA	No active or recruiting clinical trials in patients with PCa		
NY-ESO-1	Phase I trial of IMF-001 (CHP-NY-ESO-1 complex) vaccine in NY-ESO-1 expressing malignities	NCT01234012 (active study, not recruiting, estimated completion date December 2013)	NY-ESO-1 specific cellular (specific CD4 and CD8+ T cells) and humoral immunity (NY-ESO-1 antibody titer)
			Primary endpoint: safety and tolerability of the vaccine
	Phase I trial of DEC-205-NY-ESO-1 fusion protein vaccine in NY-ESO-1 expressing solid tumors	NCT01522820 (ongoing and recruiting trial, estimated completion date September 2014)	NY-ESO-1 specific cellular and humoral immunity Primary endpoint: safety of the vaccine
MAGE-A genes	No active or recruiting clinical trials in patients with PCa		
AKAP-4	No active or recruiting clinical trials in patients with PCa		
MUC-1	Phase I/II study of autologous DCs loaded with Tn-MUC-1 peptide in patients with CRPC	NCT00852007 (active study, not recruiting, estimated completion date March 2014)	Induction of CD4/CD8 responses measured by CFSE or ICS assay and/or induction of humoral response measured by specific antibodies or antibody isotype switching
			Primary endpoint: time to radiographic progression
	Phase I study of MUC-1 vaccine in conjunction with poly-ICLC in patients with recurrent or advanced PCa	NCT00374049 (active study, not recruiting, estimated completion date July 2014)	Primary endpoint: to evaluate the efficacy of poly-ICLC in boosting the immunologic response of a MUC-1 vaccine
	Phase II study of L-BLP25 (Stimuvax) in combination with androgen deprivation therapy and radiation therapy in patients with high-risk PCa. L-BLP25 vaccination is thought to work via killing of MUC-1 overexpressing cancer cells	NCT01496131 (ongoing and recruiting trial, estimated completion date January 2016)	Change in the ELISPOT level of Mucin-1-specific T cells after radiation therapy

Other PSA-expressing vectors have been tested in phase I trials in patients with PCa with rising PSA levels. Vaccinations with vaccinia-based vectors expressing PSA resulted in stabilization of serum PSA levels and PSA-specific T-cell responses were observed (34). PSA-specific T cells were also detected after vaccination with a liposome-based PSA vaccine and a dendritic cell (DC)-based vaccine (35, 36). Treatment with a PSA encoding poxviral vector vaccine in combination with radiotherapy not only showed PSA-specific T-cell activation, but also T-cell responses against prostate-associated antigens not encoded by the vaccine. This is indicative for tumor cell killing and subsequent epitope spreading (37). Hence, PSA-targeted immunotherapy can boost conventional treatment strategies to induce stronger and broader effects. This was also shown in a recent study combining PSA-TRICOM treatment with the T-cell checkpoint inhibitor ipilimumab, where the majority of chemo-naive patients displayed a decline in serum PSA levels (61).

Despite the fact that PSA-based immunotherapeutic approaches can stimulate cytotoxic T lymphocytes (CTLs) both *in vitro* and *in vivo*, untreated patients with PCa often fail to induce a potent immune response against this antigen (62–64). Several factors might contribute to this phenomenon: (i) PSA activates TGF-β, which can suppress immune responses in the tumor microenvironment (65, 66); (ii) PSA has a negative effect on lymphocyte proliferation and differentiation (63, 64); (iii) PSA can inhibit the maturation, function, and survival of DCs (63, 64).

In summary, the serine protease PSA is expressed at high levels by most PCa. Targeting PSA might not only elicit a tumor-specific immune response, but also counteract the negative effect of PSA on both T cells and DCs. Therefore, PSA poses as a promising target antigen in immunotherapy, and this is underscored by the results of phase II trials using PSA in vector-based peptide vaccines (60, 67). The ongoing phase III clinical trial (NCT01322490) might provide more evidence on the clinical relevance of PSA-TRICOM/PROSTVAC-VF vaccinations (Table 2).

### Prostate acid posphatase

Human prostate acid posphatase (PAP) is a secreted glycoprotein enzyme synthesized in the prostate epithelium (68). Only a few substrates have so far been identified for PAP, including adenosine monophosphate, phosphotyrosine, phosphocholine, phosphocreatine, and ErbB-2 (69, 70) Since PAP can act as a protein tyrosine phosphatase, many other yet to be identified substrates might be involved in the signal transduction of this protein. PAP is secreted by the prostate gland following puberty and its expression is correlated with testosterone. It is reported to enhance the mobility of sperm (71). Serum PAP levels are low in healthy individuals and increased levels are associated with PCa. For example, it is shown that PAP is aberrantly expressed in high Gleason score PCa (72, 73). Ozu et al. showed that serum PAP levels, like serum PSA, are significantly increased within the escalating PCa disease stages. PAP is also elevated in patients with bone metastasis, compared to those without bone metastasis (74). Elevation of PAP is associated with significantly shortened survival, while its decrease is correlated with responsiveness to therapy (75–77).

#### PAP as tumor antigen

Due to its elevated expression in PCa, PAP has been investigated as a possible target antigen for immunotherapeutic approaches. PAP-specific cytotoxic T cells (CTLs) can be found in blood of healthy donors and in patients with chronic prostatitis (26, 78, 79). In addition, patients with PCa vaccinated with DCs loaded with murine PAP showed responses against human PAP coinciding with significant clinical anti-tumor responses (80). Specific CTLs can also be generated by culturing with antigen presenting cells pulsed with a PAP-derived HLA-A2 binding peptide. The obtained CTLs can lyse peptide-loaded target cells in an antigen-specific manner, as well as HLA-A2 positive prostate tumor cells *in vitro* (78). PAP-specific cytolytic T-cell responses have additionally been identified in HLA-A2 transgenic mice immunized with the PAP encoding DNA vaccine pTVG-HP (81). Moreover, PAP peptides with the ability to bind additional HLA-A alleles has also been described (82, 83). Also, small clinical studies using a PAP-derived peptide for different HLA-subclasses show promising results in patients with PCa (84, 85). Naturally occurring PAP-specific CD4+ T cells are only found in 7–11% of patients with PCa, but this can be augmented by immunotherapy. Overall, these data suggest that PAP-specific T-cell responses can be initiated, and that PAP is an interesting candidate to use in cancer immunotherapy (81, 83, 84).

#### DNA-based PAP vaccine

In a PAP-based DNA vaccine, patients with CRPC received six vaccinations with granulocyte-macrophage colony-stimulating factor (GM-CSF) biweekly. Both humoral and cellular immune responses were detected in 3 of the 22 patients, with an at least threefold increase in PAP-specific IFN-gamma secreting CD8+ T cells. Nine of 22 patients showed PAP-specific CD4+ and/or CD8+ T-cell responses, but no antibody responses were detected. Also, an increase in the PSA doubling time was observed (86). The results of two ongoing trials will shed light on the role of PAP-based DNA vaccines in PCa (Table 2).

#### APC-based PAP vaccine: sipuleucel-T

After three phase III randomized controlled trials, the PAP-targeting vaccine sipuleucel-T, became the first cellular immunotherapy ever to be approved for any malignancy by the FDA (38, 39, 41). Sipuleucel-T is a peripheral blood mononuclear cell (PBMC)-based autologous vaccine. PBMCs are cocultured with a fusion protein, consisting of GM-CSF and PAP, for *ex vivo* activation of APCs and as tumor-associated antigen, respectively. The proposed mechanism of sipuleucel-T is inducing antigen-specific immune responses and thereby destroys PCa cells (40).

Sipuleucel-T treatment consists of three injections at 2-week intervals. In three phase III randomized controlled trials, an increase in overall survival of 4 months was noticed with no difference in progression-free survival. In general, treatment was well tolerated and only rigors and pyrexia were reported as adverse events (38, 39, 41). The trial by Kantoff et al. showed a trend of superior treatment outcome of sipuleucel-T in patients in the lowest PSA-level quartile (≤22.1 ng/mL). On the contrary, in the highest PSA-level quartile treatment with sipuleucel-T showed only 2.8 months overall survival benefit (41). This suggests that treatment with sipuleucel-T should be initiated directly after the diagnosis of mCRPC, when patients have a lower tumor load, hence less immune suppression.

To date, the OS benefit of sipuleucel-T cannot be fully explained by the recorded immune responses. An elevated T-cell stimulation index was observed in the sipuleucel-T treated group. Nevertheless, T-cell proliferation responses to the fusion protein (PA2024) or PAP did not show a survival difference. Increased antibody levels against PA2024 were observed in 66.2% of the sipuleucel-T treated patients and in 2.9% of the placebo-treated patients coinciding with a slight, although not significant, survival benefit (*P* = 0.08). Increased antibody levels against PAP were noticed in 28.5% of the sipuleucel-T treated patients and in 1.4% of the placebo-treated patients, not correlating with survival (41). Research is currently ongoing to define additional biomarkers that could be related to increased overall survival.

To conclude, sipuleucel-T is the first autologous cellular immunotherapy for the treatment of PCa. Three phase III trials demonstrated crucial clinical evidence for the worthiness of sipuleucel-T. However, although an increase in overall survival of 4 months is beneficial for the patients, it is not the breakthrough for immunotherapy many researchers were hoping for. Cellular immunotherapy might not be a monotherapeutic alternative for PCa. Instead, combination with standard or novel treatment modalities might be decisive. Currently ongoing trials are focusing on combination therapies with androgen deprivation therapy, chemotherapy, and immune checkpoint inhibitor antibodies (Table 2).

### Prostate-specific membrane antigen

Prostate-specific membrane antigen, also known as glutamate carboxypeptidase II, is a zinc metalloenzyme with folate hydrolase activity that is expressed in membranes of prostate epithelial cells (87, 88). Its function in the prostate is still unknown. Low expression of PSMA is also found in the kidneys, salivary glands, duodenum, and the central and peripheral nervous system.

#### PSMA as tumor antigen

Prostate-specific membrane antigen is highly overexpressed in PCa and increased expression correlates with advanced disease and metastasis (89–91). It has also been shown that PSMA is involved in tumor angiogenesis of many solid tumors, and it is expressed in the endothelial lumen in tumors. Normal vascular endothelium in non-cancerous tissue is PSMA negative (92, 93). PSMA displays several features that qualify it as a suitable target for immunotherapy. In addition to its specific expression in the prostate, it is also a membrane-bound antigen that is presented on the cell surface, but not released into the circulation (94). PSMA has been exploited as a possible target for PCa treatment in different pre-clinical settings and in early-stage clinical trials (42, 43, 88, 95). Wolf et al. showed that the recombinant anti-PSMA-specific single-chain immunotoxin D7-PE40 was both specific and highly toxic for PSMA-expressing PCa cells *in vitro* and *in vivo* in prostate tumor-bearing mice (88). Usage of the ^177^lutetium radiolabeled anti-PSMA monoclonal antibody J591 induced a 50% PSA reduction in 4 of the 35 patients with mCRPC (95). A similar PSA decrease was seen in an early clinical trial with PSMA peptide-pulsed DCs, where 9 of 33 patients displayed a partial clinical response (43). However, not all studies targeting PSMA have showed encouraging results. The PMSA-derived HLA-A2-restricted peptide (LLHETDSAV) appeared to be poorly immunogenic compared with other HLA-A2-restricted peptides, both *in vitro* as well as in patients with PCa (42). This underscores the importance of pre-clinical studies before clinical testing.

In summary, based on the highly specific expression pattern of PSMA in patients with PCa, PSMA poses as a suitable target for immunotherapy. However, early clinical trials have shown varying results. Further research concerning PSMA-based immunotherapy is warranted. Table 2 shows several ongoing clinical studies targeting PSMA as a tumor antigen.

### Prostate stem-cell antigen

Prostate stem-cell antigen (PSCA) is a glycosylphosphatidylinositol (GPI)-anchored protein expressed on the cell surface of both basal and luminal cells in the normal prostate, but overexpressed by PCa cells (44, 96). It is shown that PSCA, like other GPI-anchored proteins, is involved in the survival of stem cells, in T-cell activation and proliferation, and in cytokine and growth factor responses (97, 98). Furthermore, several studies have connected the Ly-6 family of PSCA-like GPI-anchored proteins to tumor growth and metastazation (99–102).

#### PSCA as tumor antigen

Its distinct expression pattern and possible function in tumor-progression makes PSCA an interesting target for immunotherapy. It has already been exploited in several studies, with promising results (45, 103–105). Anti-PSCA monoclonal antibodies have been reported to inhibit tumor growth and prolong the survival of mice bearing human PCa xenografts (46, 106). Additionally, a chaperone complex vaccine made of PSCA and the heat-shock protein GRP170 was shown to enhance T-cell-mediated immune responses, inhibit tumor growth, and prolong the life span of PCa tumor-bearing mice (107). Two DC vaccination studies have been performed in humans (45, 105). In the study by Thomas-Kaskel et al., patients with mCRPC were treated with DCs loaded with PSCA and PSA peptides. Endpoints were safety and induction of antigen-specific immunity. The vaccine was well tolerated in all patients, and 6 of 12 patients showed stable disease after four vaccinations. One patient had a complete response. Interestingly, this patient displayed an increase in serum PSA levels. Positive delayed-type hypersensitivity skin reactions were seen in four patients after four vaccinations. A positive delayed-type hypersensitivity test was associated with increased overall survival. HLA tetramer analysis detected high frequencies of peptide-specific T cells in one patient, who had an overall survival of 27 months (105). In another study, vaccinations were performed in three patients with mCRPC using multi-epitope (PSCA, PSMA, PAP, and PSA) pulsed DCs. The treatment was well tolerated, and significant CTLs responses against all PSAs were observed. In addition, DC vaccination was associated with an increase in PSA doubling time (45).

To conclude, PSCA has been used as a target for antigen-based immunotherapy in several clinical studies due to its role in tumor growth and metastases. Unfortunately, the study results were less impressive than expected. This might be the reason that to date there is no ongoing clinical trial with PSCA registered.

### Mucin-1

The mucin family members include proteins that enclose tandem repeat structures with a high proportion of prolines, threonines, and serines. The family consists of secreted and transmembrane forms, designated Mucin-1 (MUC-1) to MUC-21 (108). MUC-1 is a large cell surface glycoprotein found on the apical surface of most glandular and ductal epithelial cells, such as the lungs, intestines, and the prostate (109). In chronic inflammation, MUC-1 expression is induced by inflammatory cytokines like TNF-α, IFNγ, and IL-6. Overexpression contributes to oncogenesis by activation of growth and survival pathways (Wnt-β-catenin and nuclear factor-κB pathways), promoting receptor tyrosine kinase signaling and downregulation of stress-induced death pathways (108). MUC-1 overexpression is associated with colon, breast, lung, prostate, and pancreatic cancer. Moreover, it is associated with tumor-progression and correlated with advanced disease (110–112). MUC-1 has also been shown to have immunosuppressive effects in mice, and secreted MUC-1 has been shown to block T-cell activation (113, 114). Moreover, human monocyte-derived DCs cultured *in vitro* with MUC-1 peptide displayed a decreased expression of both co-stimulatory molecules and antigen presenting molecules upon activation (115). Similarly, depletion of soluble MUC-1 in tumor cell line supernatants abolished the anti-proliferative effect of these supernatants on T cells, and MUC-1 has therefore been identified as a target in PCa (116). The inhibitory effect of MUC-1 has also been demonstrated *in vivo*, when synthetic MUC-1 decreased the immune response in patients vaccinated with an MUC-1 containing polyvalent peptide vaccine (117). In a recent phase I/II trial, an autologous DC vaccine loaded with an MUC-1 glycoproteine and KLH in patients with CRPC was studied. Patients received three injections biweekly followed by booster vaccinations at 6 and 12 months. The rate of PSA rise decreased in six of seven patients. The PSA doubling time increased from a median of 2.9 months prior to vaccination to 7.5 months during vaccination (118). Richman et al. also showed clinical benefit for some patients with mCRPC treated with the combination of radioimmunotherapy with an anti-MUC-1 monoclonal antibody and paclitaxel (47) (Table 1).

Taken together, MUC-1 is important in tumor-progression and therefore a very interesting tumor-associated antigen. Several trials focusing on MUC-1 as a target for cancer immunotherapy in PCa are ongoing (Table 2).

### Cancer/testis antigens

Cancer/testis antigens are normally only expressed in gametogenic tissue. However, this group of proteins is aberrantly expressed in several types of cancers, including PCa (48). CTAs have been shown to contribute to tumor formation and progression (119, 120). The CTAs NY-ESO-1, the MAGE family, and A-kinase Anchor Proteins (AKAP)-4 will be discussed here.

NY-ESO-1 is found to be expressed in a variety of malignancies. It is not expressed in normal adult tissue, with the exception of the testis. The expression of NY-ESO-1 is associated with level of disease, and higher NY-ESO mRNA and protein expression are observed in metastatic and advanced PCa, as compared to localized tumors (120–124). The function of NY-ESO-1 is unknown, but it is speculated to play a role in meiosis or in the assembly of the organelles that develops over the anterior half of the head in the spermatozoa (125, 126). The NY-ESO-1 is a promising candidate because of its tumor-restricted expression and the identification as one of the most immunogenic CTAs, eliciting spontaneous cytotoxic and antibody-mediated immune responses in patients with NY-ESO-1 + tumors (127–129). Humoral responses against NY-ESO-1 have been evoked by non-specific immune activation in patients with mCRPC treated with a combination of checkpoint inhibitor ipilimumab and GM-CSF, underscoring its immunogenicity (130). NY-ESO-1 has been used as target antigen in several clinical studies. Both MHC class I and II restricted T-cell epitopes specific for NY-ESO-1 are identified (131). MHC class I and/or II restricted NY-ESO-1 peptides were compared in a peptide-based vaccine trial in patients with mCRPC. The vaccine increased the PSA doubling time and yielded antigen-specific T-cell responses in all patients treated. The strongest results were seen in chemo-naive patients, most likely due to a lower tumor burden, thus less tumor-induced immune suppression (132). The immunogenic features of NY-ESO-1 are further supported by a study using a protein-based vaccine with CpG as an adjuvant. This vaccine was able to prime antigen-specific B-cell responses and induced NY-ESO-1 specific, tumor-reactive CTLs in patients with metastatic PCa, independently of autologous NY-ESO-1 expression (49). Vaccination against a tumor-specific protein without it being present, repositions this clinical vaccination protocol toward a preventive setting.

Second, the MAGE CTA subfamily is also expressed in PCa. Upregulation of these CTAs is found in CRPC and is associated with resistance to chemotherapeutic agents (50). MAGE-A2 downregulates p53 transactivation function through histone deacetylase recruitment, a possible explanation how MAGE-A2 expression leads to resistance to chemotherapy (50). Indeed, silencing of MAGE-A2 increased sensitivity to docetaxel chemotherapy in PCa tumor cells (120). Expression of MAGE-C2/CT10, another member of the MAGE-A subfamily, is correlated with the degree of PCa malignancy. It is an indication of higher risk for biochemical recurrence after radical prostatectomy and represents a potential target for immunotherapy (133). Members of the MAGE-A subfamily and NY-ESO-1 are often co-expressed in prostate malignancies.

Third, the CTA AKAP are a family of scaffolding proteins capable of controlling intracellular signals. AKAP is involved in cytoskeletal regulation and organization by affecting cyclic AMP-dependent protein kinase-A (134). In the prostate epithelium, the anchor proteins synthesize and secrete calcitonin. It has been shown that the calcitonin secretion from malignant prostates is several-fold higher than from benign prostates (135). The calcitonin receptor is expressed in malignant PCa, and its activation stimulates growth of PCa cells via activation of cyclic AMP as well as protein kinase C (136, 137). These mechanisms suggest a marked increase in the invasiveness of PCa cells (138). Modulation of protein kinase-A activation possibly interferes with the growth, tumor genicity, and metastatic potential of advanced tumors. First, AKAP-4 has been showed to be an immunogenic CTA in patients with multiple myeloma (139). Later, Chiriva-Internati et al. showed cytoplasmic and surface expression of AKAP-4 in the LnCAP PCa cell line. AKAP-4 expression in the prostate epithelial cells was shown in 13 of 15 patients with PCa, but not in healthy subjects. Cytotoxicity assays showed that AKAP-4-loaded DC-stimulated T cells were capable of killing autologous PCa cells *in vitro*. Neither killing of AKAP-4 negative PCa cells nor normal prostate epithelial cells was observed. This underscores the antigen specificity of the response and prevention of autoimmune reactions (140). This makes AKAP-4 a very interesting target for PCa anti-tumor vaccination.

To conclude, several CTAs, especially NY-ESO-1, the MAGE-A subfamily, and AKAP-4, could serve as therapeutic targets in the fight against PCa (120, 122, 140). Especially NY-ESO-1 is of major relevance in PCa and a target in different ongoing trials (see Table 2). Due to the tumor-restricted expression of CTAs, these antigens can also be used in an adjuvant or a preventive setting hindering the recurrence of CTA-positive tumors (49).

## Mixture of Tumor-Associated Antigens

To date, many investigators underscore the importance of a personalized approach by selecting patient-specific mutations as target antigens for immunotherapy. The group of Noguchi took a first step in a personalized direction. They performed two phase II studies with a personalized peptide vaccine (PPV). The vaccine consisted of four peptides based on each patient’s immunoreactivity profile. Peptides of a variety of tumor-associated antigens were tested, including PSA, PAP, PSMA, multidrug resistance protein, and a choice of different epithelial tumor antigens. The peptides included in the vaccine were selected on their capacity to induce CTL responses. In the first phase II trial, patients with CRPC were randomized to PPV combined with chemotherapy or chemotherapy only (141). Antibody responses were seen in 64% of the patients and cytotoxic T-cells responses in 72% of the patients. An increase in progression-free survival was observed in the PPV/chemotherapy group as compared with the patients who only received chemotherapy. However, immune responses did not correlate with clinical outcome in patients treated with PPV and chemotherapy. Interestingly, the authors found that lower levels of IL-6 before PPV vaccination were favorable for overall survival. IL-6 have been associated with more aggressive cancer progression and decreased survival in PCa (142). In this perspective, IL-6 may be seen as an indicator of prognosis and a predictor of therapy effectiveness. It is also hypothesized that inhibiting IL-6 signaling may be beneficial in patients enduring other immunotherapeutic treatment.

The results of the PPV vaccinations are promising. A randomized trial with an appropriate control group before and after chemotherapeutic treatment is needed to fully identify a clinical benefit of PPV treatment. Currently, a vaccine consisting of 20 peptides is applied to patients with CRPC in an exploratory, randomized, open-label study (UMIN000008209, Table 2).

## Prostate Cancer Cell Lines

### GVAX

GVAX is an allogeneic, cell-based immunotherapy consisting of the PCa cell lines LNCaP and PC-3. These cell lines are genetically modified with a recombinant GM-CSF adeno-based viral vector and irradiated before administration. Clinical results in patients with PCa are indicative for a favorable clinical outcome with no toxicities (143, 144). These results have led to phase III trials, using the most promising high-dose GVAX protocol. Unfortunately, due to an even increased mortality in the GVAX-treated group, and disappointing interim results the trials were abrogated (145, 146).

van den Eertwegh et al. combined the immune checkpoint inhibitor ipilimumab (anti-CTLA-4) with GVAX (147). More than 50% decline in PSA level was seen in 25% of the patients. All the responding patients got 3.0 or 5.0 mg/kg ipilimumab. There was dose-limiting toxicity in the 5.0 mg/kg group of patients, while the lower ipilimumab regimens were well tolerated. Markedly, all patients with immune-related adverse events showed a decrease in PSA levels. A small number of patients additionally displayed an anti-PSMA antibody response. These patients had a significant increase in median overall survival (46.5 months compared to 20.6 months for patients without this humoral response). T-cell monitoring studies were performed in 28 patients receiving the combination therapy of GVAX and ipilimumab. Compared with the control group, an increase in absolute lymphocyte counts and enhanced CD4+ and CD8+ T-cell differentiation was observed. These immune responses were associated with a significantly prolonged overall survival. In addition, an OS benefit was also seen in case of high pre-treatment levels of CD4+, CTLA-4+, CD4+/PD-1+, or non-naïve CD8+ T cells. Low pre-treatment frequencies of differentiated CD4+ or regulatory T cells resulted in a prolonged OS (148). This reveals perspectives for future biomarker research.

### mRNA-transfected DCs

An alternative approach for PCa cell lines is the use of PCa cell line-derived RNA or tumor antigen encoding mRNA. Kyte et al. transfected monocyte-derived DCs with mRNA derived from the PCa cell lines LnCAP, DU-145, and PC-3. Although the generation of mRNA-transfected DC is challenging, DC vaccination appeared feasible and safe (149, 150). Furthermore, PSA-specific T-cell responses were detected in 12 of 19 patients with PCa who underwent mRNA-DC vaccination (149). To date, patients are recruited in a phase I/II trial (NCT01197625, Table 2), studying the mRNA-transfected DCs in curative resected patients with PCa. More studies are needed to properly determine the strength of mRNA-transfected DCs. The usage of this immunotherapeutic modality within combination therapies might be of greater significance.

## Discussion

In this review, we provided overview of PCa tumor-associated antigens and how they are used to target PCa via immunotherapy (Table 1). PSMA and PSCA are normally expressed in the prostate gland but upregulated during cancer development and they may play a role in tumor progression (44, 89, 96, 151). Increased serum levels of secreted tumor antigens, such as PSA and PAP, can be used as biomarkers for disease and disease progression (51, 73, 74). More general tumor antigens, like MUC-1, AKAP-4, and NY-ESO-1, can also be found in PCa and might be candidates for immunotherapeutic interventions (111, 123, 140). MUC-1 is expressed in normal tissue and upregulated on several tumors, where it can exert immunosuppressive effects and attain tumor growth (110). Hence, targeting MUC-1 could have a dual role – directing the immune response toward the tumor and reducing immune suppression. This might also be valid for other immunosuppressive antigens, such as the MAGE-A subfamily or PSA (54, 120). On the other hand, the NY-ESO-1 antigen is often immunogenic *per se*, and pre-existing immune responses directed against this antigen are common in treatment-naive patients (128). Pre-existing CTL responses against PSA and PAP in healthy individuals and patients with chronic prostatitis also support the definition of PCa as an immunogenic tumor (26, 27), where tolerance against self-antigens can be broken and the immune system can be harnessed against the tumors.

Today, the only registered product for antigen-targeted immunotherapy in PCa is sipuleucel-T (38, 39, 41). Although the significance of this intervention received criticism, sipuleucel-T proves an important point: autologous cellular immunotherapy is feasible and can indeed be developed as an approved treatment modality. To date, no convincing mechanism of action has been elucidated for sipuleucel-T. Increased immune responses were observed but no correlation with clinical outcome could be established. Clinical studies aiming at identifying immunological responses and thereby hopefully providing an in-depth understanding of the mode of action of sipulecuel-T are ongoing. Unraveling the mechanism might be beneficial for further development of sipuleucel-T and other immunotherapeutic approaches.

Effective immune responses induced by immunotherapeutic treatments are still not common, and probably vary depending on tumor type, somatic differences between tumor cells, and the tumor microenvironment (66). Several recent trials have shown promising results in both clinical and immunological responses. Constructs targeting the NY-ESO-1 antigen has led to significant immunological responses, which makes NY-ESO-1 an interesting antigen to target immunotherapeutic strategies in future (49, 132). Immunological responses are also induced by several PSA-targeting vaccines, supporting the usage of PSA as an immunogenic tumor antigen (34–36).

Insight in the localization of the tumor antigen (on/in cells, normal cells vs. tumor cells, in organs) and the specificity of the antigen facilitates a precise selection of target antigens with the intention of optimizing the translation of immunotherapeutic treatments to the clinic. However, despite significant T-cell responses, tumor progression is seen most frequently in patients treated with cancer immunotherapy. This is due to the complexity of human beings and the complexity of tumors and metastases (152). The complexity of cancer is also described by Fox et al. (153). This report of the collaborating immunotherapy organizations, known as the Society for Immunotherapy of Cancer (SITC), contains the identification of nine hurdles in cancer immunotherapy that significantly delays clinical translation of promising cancer immunotherapeutics. We here discuss the hurdles relevant for this review, for a complete overview, we refer to the original article (153). The first hurdle to overcome is the complexity of cancer, tumor heterogeneity, and immune escape. The immune signature of the tumor, distinguished by genetic or histological evaluation, can predict responders to cancer immunotherapy (154, 155). The second relevant SITC hurdle for this review is the lack of definitive biomarkers for the assessment of clinical efficacy of cancer immunotherapies. Biomarkers to distinguish between patients responsive to initial treatment, patients displaying immune inhibitory features, and patients with non-immunogenic tumors, are needed. Pre-existing anti-tumor responses or the expression of inhibitory markers are examples of suggested biomarkers that could be used to predict treatment outcome and individualize the treatment regime.

A correlation of immune parameters with clinical outcome after immunotherapy is not established in patients with PCa. This can be attributed to (i) a limited number of patients per immunotherapeutic approach; (ii) a variation in clinical features of patients with PCa before treatment; and (iii) the difference in clinical signs of tumor control between conventional toxic treatments and immunotherapeutic treatments. This last argument is also one of the hurdles identified by the SITC. Effective immunotherapy does not always display initial shrinkage of the tumor, but rather a pattern of tumor growth and progression followed by shrinkage when the tumor is recognized and destroyed by the immune system (156–158). This paradox has been illustrated by the negative outcomes on progression-free survival or PSA responses in the sipuleucel-T trials and PSA-TRICOM trial. Tumor swelling, increased release of PSA due to elevated tumor cell death, and initial detrimental symptoms might be associated with a favorable clinical outcome rather than with progressive disease, as stated in the WHO and Response Evaluation Criteria In Solid Tumors (RECIST) criteria (156, 157, 159). Although clinically responding patients might have been missed, some patients do not respond, neither clinically nor immunologically. Lack of immunogenicity of the antigens used might be an explanation, but a major factor is the immunosuppressive networks within cancer patients. Infiltrating lymphocytes can be regulated by a number of inhibitory pathways within the tumor and thereby shift the direction of the ongoing immune response toward a more tolerogenic one. Other patients might have “silent” tumors that do not display an inflammatory phenotype and hence do not attract lymphocyte infiltration (66).

Novel monoclonal antibodies targeted against inhibitory receptors on T cells (anti-CTLA-4 and anti-PD-1) are able to prolong their effector functions and prevent immune inhibition. These treatment strategies are tested in combination with other immunotherapeutic approaches and showed promising results in a subset of patients (61, 147, 160). There are still ongoing combination therapy studies with ipilimumab and sipuleucel-T which will hopefully overcome the immunosuppressive signals provided by immune evading tumors (NCT01804465 and NCT 01832870). Although antigen-based immunotherapy itself seldom gives rise to severe autoimmune reactions, the combination with an immune checkpoint inhibitor likely will enhance the risk of immune-related adverse events, as recently shown in melanoma patients treated with ipilimumab (161–163).

## Future Challenges and Opportunities

Until recently, patients with mCRPC had limited treatment options and a poor prognosis. With new sequential hormonal therapies, second-line chemotherapy, and new immunotherapeutic strategies, a new era has started. To date, PCa is one of the few tumor types in which immunotherapy is part of the current standard of care. Augmenting immune responses to PCa antigens is a valid therapeutic approach, and clinical responses with minimal toxic effects are observed.

In this review, we focused on commonly expressed tumor-associated antigens. Recently, patient-specific epitopes are identified as highly important to improve T-cell reactivity. Targeting these patient- and cancer-specific mutated epitopes holds promise for even better results and possibly cure of patients. By complete genome and transcriptome, sequencing and mass spectrometry-mutated HLA-binding peptides, so-called neoantigens, might be identified (164). Vaccination with individually overexpressed tumor-specific peptides could result in a unique, personalized anti-cancer vaccine (165–167). Recently, the first two demonstrations of autologous cancer exome-based T-cell responses against patient-specific neoantigens in humans were published (167, 168). This knowledge is a major step forward for both the identification of new diagnostic strategies by tumor exome analysis, as well as for the development of individualized immunotherapeutic approaches. Combination therapies harboring these patient-specific peptide vaccinations together with immune checkpoint inhibitors are likely to generate an even better immune control. There is no doubt that these are very exciting times for cancer immunotherapy.

## Conflict of Interest Statement

Harm Westdorp, Annette E. Sköld, Berit A. Snijer, Sebastian Franik, Sasja F. Mulder, Pierre P. Major, Ronan Foley, and I. Jolanda M. de Vries declare no disclosures of potential conflicts of interest. Winald R. Gerritsen is a consult for Aglaia Biomedical Ventures, is on the supervisory board for PsytoBe, has received speakers fee from J&J and BMS, and is on the advisory boards for Amgen, BMS, Janssen-Cilag, Sanofi, Astellas, Bayer, and Merck.
